# Clarification of Orange Press Liquors by PVDF Hollow Fiber Membranes

**DOI:** 10.3390/membranes6010009

**Published:** 2016-01-20

**Authors:** Silvia Simone, Carmela Conidi, Claudia Ursino, Alfredo Cassano, Alberto Figoli

**Affiliations:** Institute on Membrane Technology, ITM-CNR, via Pietro Bucci, 17/C, I-87036 Rende (CS), Italy

**Keywords:** orange press liquor, microfiltration, hollow fiber membrane, polyphenols

## Abstract

Press liquors are typical by-products of the citrus juice processing characterized by a high content of organic compounds and associated problems of environmental impact, which imply high treatment costs. However, these wastes contain a great number of health promoting substances, including fibers, carotenoids and phenolic compounds (mainly flavonoids), whose recovery against waste-destruction technologies is very attractive for new business opportunities. In this work, the clarification of orange press liquor by using microfiltration (MF) membranes is studied as a preliminary step to obtain a permeate stream enriched in antioxidant compounds which can be further processed to produce extracts of nutraceutical and/or pharmaceutical interest. MF poly(vinylidene fluoride) (PVDF) hollow fibers were prepared by the dry/wet spinning technique. A series of fibers was produced from the same polymeric dope, in order to investigate the effect of selected spinning parameters, *i.e.*, bore fluid composition and flowrate, on their properties. The morphology of the produced fibers was analyzed by Scanning Electron Microscopy (SEM). Fibers were further characterized for their mechanical properties, porosity, bubble point, pore size distribution and pure water permeability (PWP). Some of the produced fibers exhibited high permeability (pure water permeability ~530 L/m^2^·h·bar), coupled to good mechanical resistance and pore size in the range of MF membranes. These fibers were selected and used for the clarification of press liquor from orange peel processing. In optimized operating conditions, the selected fibers produced steady-state fluxes of about 41 L/m^2^·h with rejections towards polyphenols and total antioxidant activity of 4.1% and 1.4%, respectively.

## 1. Introduction

Citrus is one of the world’s major fruit crops largely developed in tropical and sub-tropical countries. The annual global production of citrus fruits was estimated of over 115 million metric tons in 2011 with oranges contributing more than half of the worldwide citrus production. According to 2012 data from the Food and Agriculture Organization of the United Nations (FAO), China, Brazil, the U.S.A., India, Mexico, and Spain are the world’s leading citrus fruit-producing countries, representing close to 60% of the global production [1].

The citrus juice production is accomplished by a huge amount of waste material such as peels and seeds. These wastes are traditionally spread on soil producing dried peel by natural evaporation which can be used as swine or cattle feed [2]. This method of handling presents environmental and health problems due to uncontrolled fermentation and produced leachates containing high concentrations of organic matter which can contaminate surface and ground waters. An alternative handling treatment of citrus peels involve the use of lime followed by milling and pressing. The resulting press liquor, with an average total soluble solids (TSSs) content of 10 °Brix, can be concentrated up to 65–70 °Brix by multiple effect evaporation to obtain citrus molasses which can be used in the production of beverage alcohol and as cattle feed. Reverse osmosis (RO) and forward osmosis (FO) have been proposed recently as alternative systems to thermal evaporation [3,4]. As a by-product of the orange juice production, the press liquor is enriched in bioactive compounds, such as flavonoids and phenolic acids, recognized for their beneficial implications in human health. Epidemiological studies have correlated their consumption with a lower risk of different types of cancer and cardiovascular diseases thus showing that they possess antioxidant, anti-inflammatory and radical-scavenging activity [5,6,7].

The valorization of these agro-food wastes has the double advantage of reducing the pollution load and contributing to sustainable development through the rational use of natural raw materials. In particular, the recovery of high-added-value compounds against current waste-destruction technologies suggests new business opportunities in the formulation of products of interest in food (dietary supplements and functional foods production), pharmaceutical (products with antibacterial, antiviral, anti-inflammatory, antiallergic and vasodilatory action) and cosmetic industry [8].

Over the past years, different studies have been proposed for the recovery of phenolic compounds from by-products of orange juice processing based on the use of organic solvents, resins, heat treatment, γ-irradiation and enzymes [9,10,11,12,13]. However, the proposed methodologies are characterized by several drawbacks. For example, the extraction with organic solvents presents safety problems (some of them are believed to be toxic), low efficiency and time consumption; heat treatment results in pyrolysis; γ-irradiation assisted extraction is still unknown in terms of safety.

Membrane processes are widely accepted as viable alternatives to traditional techniques; several processes are worldwide actually implemented on a large scale in several fields. It is generally recognised that membranes processes bring about several advantages, since they do not usually involve phase changes, allow reduction of chemical additives use, are simple in concept and operation, modular and easy to scale up. Moreover, they require low energy consumption and show great potential to be coupled also to alternative energy sources. All of these advantages translate into cost savings and more environmentally sustainable processes in agreement with the strategy of process intensification [14].

In recent years, membrane processes have spread over into the food industry as innovative techniques. For example, microfiltration (MF) is widely applied to the treatment of wine and milk, and, in general, of all the liquid media that need to be cleansed of undesirable particles such as bacteria or other harmful microorganisms, which adversely affect their hygiene and safekeeping. This process has been proposed as macroscopic pre-treatment within the well-established “5-Stages Universal Recovery Processing” strategy for the recovery of valuable compounds from food wastes [15,16].

Among all the possible configurations, hollow fiber (HF) membrane modules are usually preferred on industrial scale since they are self-supported, more flexible and easy to handle, ensuring high surface to volume area. This translates into space-savings, more productivity and reduction of costs also connected to their maintenance, since these modules can be back-flushed [17]. Several applications of HF membranes in the treatment of agro-food products and by-products have been also investigated including the pre-treatment of olive mill wastewaters [18], the removal of pesticides from citrus essential oils [19] and the clarification of several fruit juices such as mandarin [20], kiwifruit [21], pomegranate [22], cactus pear [23] and bergamot [24] juices.

In this work, poly(vinylidene fluoride) (PVDF) HF membranes have been prepared, characterized and used for the clarification of orange press liquor. PVDF was selected among the several available polymers due to not only its good hydrophobicity and excellent chemical stability, but also the feasibility of forming hollow fibers and membranes via a phase inversion method. The performance of some selected HF membranes was evaluated in terms of productivity (permeate fluxes) and rejection towards phenolic compounds in order to produce a permeate stream suitable to be further processed for formulations of nutraceutical or pharmaceutical interest.

## 2. Materials and Methods

### 2.1. Hollow Fiber Membrane Preparation and Post-treatment

PVDF HF membranes were prepared by the dry/wet spinning technique described elsewhere [25,26,27]. The polymeric dope was prepared by dissolving poly(vinylidene fluoride) Solef® 6010 (kindly provided by Solvay Advanced Polymers, Bollate, Italy) 25 wt.% in dimethyl formamide (DMF) (Carlo Erba Reagenti, Italy). Poly-vinyl pyrrolidone Luviskol K-17 (PVP K-17, Mw = 12 kg/mol) was purchased from BASF and used as pore forming additive, in concentration of 35 wt.%. PVP was desiccated at 50 °C under vacuum overnight before use. Different fibers types were produced by varying the bore fluid composition and flow rate. All the other spinning parameters were kept constant. The detailed conditions of fiber spinning are resumed in Table 1.

**Table 1:** Detailed spinning conditions of the poly(vinylidene fluoride) (PVDF) hollow fibers produced in this work.

Dope composition (wt.%)	PVDF/DMF/PVP K-17 25/40/35
Dope flow rate (g/min)	12
Dope temperature (°C)	115
Bore fluid composition (wt.%) and flow rate (mL/min)	Fiber A: DMF 45%, 9 mL/min Fiber B: DMF 45%, 12 mL/min Fiber C: DMF 45%, 15 mL/min Fiber D: DMF 45%, 18 mL/min Fiber E: EtOH 45%, 18 mL/min Fiber F: EtOH 45%, 15 mL/min
Bore fluid temperature (°C)	50
Outer coagulant	Tap water at room temperature
Air gap (cm)	25
Spinneret dimensions (cm)	O.D./I.D. 1.6/0.6
Post treatment	NaClO 4000 ppm pH 7 overnight

Abbreviations and symbols list: PVDF = Poly(vinylidene fluoride), PVP = Poly-vinyl pyrrolidone, DMF = Dimethyl formamide, EtOH = Ethanol.

The produced PVDF hollow fibers were kept in frequently refreshed water bath for one day, to remove residual solvent and ensure complete coagulation. Afterwards, they were treated with sodium hypochlorite 4000 ppm, buffered to pH 7, in order to leach out PVP from membrane pores and increase permeability. Fibers were soaked in a glycerol/water solution (30 wt.%), in order to avoid collapse of their porous structure, before the final drying.

### 2.2. PVDF Hollow Fibers Characterization

#### 2.2.1. Morphology

The morphology of PVDF HF membranes was analyzed by Scanning Electron Microscopy (SEM) (Quanta FENG 200, FEI Co., Hillsboro, Oregon USA). Samples were broken in liquid nitrogen, in order to obtain a clean fracture of their cross section. Pictures were acquired in low-vacuum mode, and no gold sputtering was necessary prior to SEM observation.

#### 2.2.2. Mechanical Properties

The Young’s modulus (Em), tensile stress at break (Rm) and elongation at break (%) of the produced fibers were measured by a ZWICK/ROELL Z 2.5 test unit, as described elsewhere [25,26,27]. Samples were stretched unidirectionally with 5 mm/min rate; the initial distance between the clamps was 5 cm. The reported values were calculated as average of five measurements.

#### 2.2.3. Void Fraction

Fiber porosity was calculated by measuring the ratio between the volume of the pores by the total membrane volume, usually defined as void fraction, by a gravimetric method widely described in literature [25,26,27,28]. For each fiber type, three dry specimens, not treated with glycerol, were weighed with a precision balance. Fibers were, then, kept immersed in kerosene for 24 h and weighed again. The average void fraction was calculated according to the following equation:

(1) $$\varepsilon m(\%)=\frac{\frac{w_{1}-w_{2}}{D_{k}}}{\frac{w_{1}-w_{2}}{D_{k}}+\frac{w_{2}}{D_{pol}}}\cdot 100$$

where *w_1_* is the weight of the wet membrane, *w_2_* is the weight of the dry membrane, *D_k_* is the kerosene density (0.82 g/cm^3^), and *D_pol_* is the PVDF density (1.78 g/cm^3^).

#### 2.2.4. Bubble Point and Average Pore Size

Fibers bubble point and average pore size were measured by using a capillary flow porometer (CFP 1500 AEXL, PMI porous materials Inc., USA), as described elsewhere [25,26,27]. Tests were carried out according to the wet up/dry up method using Porewick (16 dyne/cm) as wetting liquid.

#### 2.2.5. Pure Water Permeability

The hydraulic permeability of the produced fibers was evaluated before experiments with orange press liquor. Fibers pure water permeability (PWP) was calculated from the slope of the straight lines fitted in the plot reporting the relationship between transmembrane water flux (J) and transmembrane pressure (TMP).

J (expressed in L/m^2^·h) was calculated by using the following equation:

(2) $$J=\frac{Q}{t\cdot A_{s}}$$

where *Q* is the amount of permeate collected at the end of each test (L), *A_s_* is the active membrane surface calculated on the basis of fibers inner diameter, length and number (m^2^), *t* is the operating time (h).

Experiments were carried out using lab-made modules, each containing three hollow fibers (20 cm length). Before any test, fibers were washed with double distilled water, at 50 °C, for 15 min, at a transmembrane pressure (TMP) of 1.25 bar, for glycerol removal. Tests were carried out working in cross-flow configuration using the inside out configuration (feed solution in fibers lumen), at a temperature of 25 °C and a flow rate of 30 L/h.

### 2.3. Orange Press Liquor

The press liquor from orange peel was supplied by Citrech Snc (Messina, Italy). It was stored at −17 °C and defrosted at room temperature before use. Before the clarification process, the liquor was filtered through a cotton fabric filter.

### 2.4. Experimental Set-Up and Procedures

Orange press liquor was clarified by using an MF bench plant (DSS Lab Unit M 10) supplied by Danish Separation System (Nakskov, Denmark) equipped with a HF membrane module.

The equipment consists of a feed tank, a cross-flow pump (ECO type GA4-KDT-TTU), two manometers located at the inlet (*P_in_*) and outlet (*P_out_*) of the membrane module, a pressure control valve and a multitube heat exchanger fed with tap water.

Experimental runs were performed in total recycle configuration recycling both permeate and retentate streams in the feed tank in order to evaluate the effect of TMP and feed flow rate on the permeate flux. The TMP value was modified in the range 0.3–0.9 bar, while the feed flow rate was modified in the range 616–1200 mL/min at a constant temperature value (25 °C).

The performance of HF membranes in terms of productivity and selectivity towards biologically active compounds was also evaluated in experimental runs performed according to the batch concentration configuration (recycling the retentate stream in the feed tank and collecting the permeate separately), in optimal operating conditions (TMP, 0.6 bar; T, 25 °C; feed flow rate = 1200 mL/min).

The fouling index (*FI*) of HF membranes was calculated by comparing the pure water permeability before and after the treatment of orange press liquors based on following equation:

(3) $$FI=(1-\frac{PWP_{1}}{PWP_{0}})\cdot 100$$

where *PWP_0_* and *PWP_1_* are the pure water permeability before and after the press liquor filtration.

### 2.5. Analytical Methods

Feed, permeate and retentate samples collected during the clarification process were analyzed for total soluble solids (TSS), pH, total suspended solids, total antioxidant activity (TAA), total polyphenols and flavonoids. The rejection (*R*) of the membranes towards the investigated compounds was determined as:

(4) $$R=(1-\frac{C_{p}}{C_{f}})\cdot 100$$

where *C_p_* and *C_f_* are the concentration of a specific component in the permeate and feed, respectively.

#### 2.5.1. Total Soluble Solids, pH and Total Suspended Solids

TSS measurements were carried out by using a hand refractometer (Atago Co., Ltd., Tokyo, Japan) with scale range of 0–32 °Brix. pH was measured by an Orion Expandable ion analyser EA 920 pH meter (Allometrics, Inc., Baton Rouge, LA, USA). The suspended solids content was determined in relation to total juice (w/w, %) by centrifuging, at 2000 rpm for 20 min, 45 mL of a pre-weighted sample; the weight of settled solids was determined after removing the supernatant.

#### 2.5.2. Total Antioxidant Activity

TAA was determined by an improved version of the 2,2’bis-azino-(3-ethylbenzothiazoline-6-sulfonic acid) diammonium salt (ABTS) free radical decoluration assay in which the radical is generated by a reaction with potassium persulfate before the addition of the antioxidant [29,30]. This method gives a measure of the antioxidant activity of pure substances and of mixtures by monitoring the reduction of the radical cation as the percentage inhibition of absorbance at 734 nm. Spectrophotometric measurements were performed by using a UV–Visible recording spectrophotometer (UV-160 A, Shimadzu Scientific Instruments, Inc., Japan) at 30 °C.

#### 2.5.3. Total Phenol Content

Total phenols were estimated colorimetrically by using the Folin–Ciocalteu method [31]. The method is based on the reduction of tungstate and/or molybdate in the Folin-Ciocalteu reagent by phenols in alkaline medium resulting in a blue colored product (max 756 nm). Results were expressed as mg/L gallic acid.

#### 2.5.4. Determination of Flavonoids

Flavonoids (hesperidin, naringin and neohesperidin) were determined by using an HPLC system (Agilent 1100 Series, USA) equipped with a pump, a UV-Vis detector and a data acquisition system. Chromatographic separation was performed by using a Luna C 18(2) column (250 mm × 4.6mm, 5 m, Phenomenex, Torrance, CA, USA); the following conditions were used: flux, 1 mL/min; T, 25 °C; pressure, 100 bar; 284 nm. The mobile phase was a mixture of 80:20 water/KH_2_PO_4_ 0.25M(v/v) (solvent A) and a mixture of 46:4:50 water/KH_2_PO_4_ 0.25 M/acetonitrile (solvent B). A six-step linear gradient analysis for a total run time of 40 min was used.

The identification and quantification of flavonoids in orange press liquor juice was based on the external standard method by comparing the retention times and their UV-Vis spectra with those of representative standards at different concentrations.

Working solutions of hesperidin, naringin and neohesperidin were prepared by dissolving commercial flavonoids standards (Extrasynthese, Genay, France) in ethanol (Sigma Aldrich, Milan). Samples of orange press liquor were dissolved in a small quantity of DMSO (2 mL) and 10 mL of ethanol were added later. The obtained solutions were filtered with 0.45 m cellulose acetate filters and directly injected.

#### 2.5.5. Statistical Analysis

Analyses of physicochemical parameters were performed in triplicate. Results were given as mean ± standard deviation. One-way analysis of variance (ANOVA) was used to compare the means. Differences were considered to be significant at *p* < 0.05. Statistical analyses were performed with use of Microsoft Excel software (version 2010; Microsoft Corporation; Redmond, WA, USA).

## 3. Results and Discussion

### 3.1. Hollow Fiber Morphology

**Figure 1:**
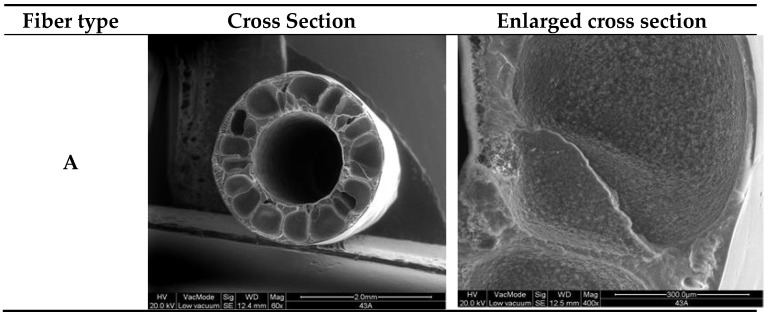

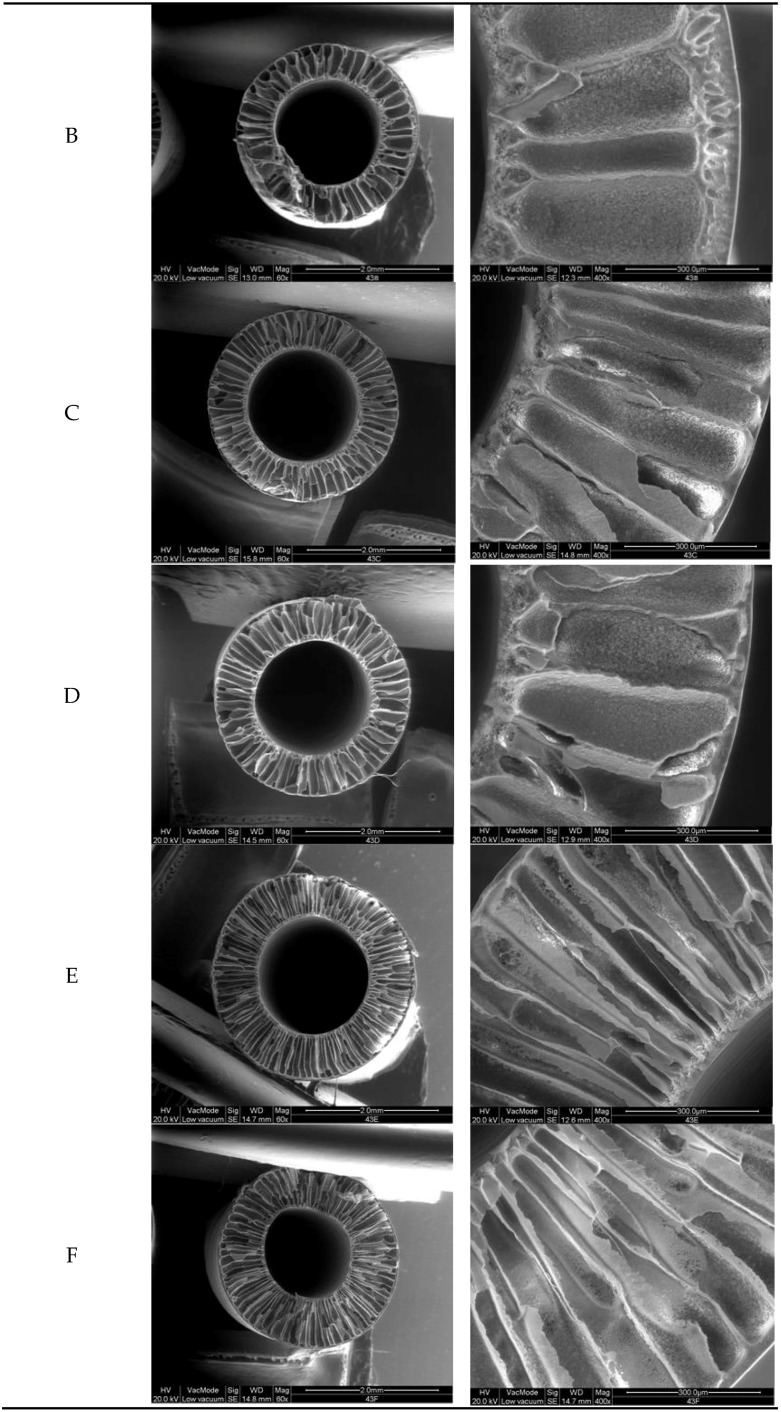
SEM pictures of the PVDF hollow fibers produced using the indicated bore fluid composition and flow-rate: A) DMF 45%, 9 mL/min; B) DMF 45%, 12 mL/min; C) DMF 45%, 15 mL/min; D) DMF 45%, 18 mL/min; E) EtOH 45%, 18 mL/min; F: EtOH 45%, 15 mL/min.

As reported in Table 1, six different fiber types were spun from the same dope, varying the bore fluid composition and flowrate. The SEM pictures of the produced PVDF hollow fibers are reported in Figure 1. All fibers exhibited a symmetric structure, mainly made up of parallel finger-like macrovoids. The predominance of finger-like macrovoids, observed in the produced PVDF hollow fibers, can be explained taking into account the balance between thermodynamic and kinetic factors during coagulation. As widely accepted in literature [32], the morphology of membranes produced via phase inversion depends on a trade-off between kinetic and thermodynamic factors. The first category includes viscosity of the dope; viscosity, dimensions and surface tension of solvent and non-solvent molecules, which affect their inter-diffusion. The second category comprises mutual affinity (Hildebrand’s parameters) between polymer, solvent, non-solvent, and temperature. These factors may hinder or enhance L/L demixing during membrane formation, thus affecting membrane morphology, in particular, promoting or avoiding macrovoid development.

Lee *et al.* [33] investigated the trade-off between thermodynamic and kinetic factors, in polysulphone (PSU) membranes formation, using the additive PVP. They observed that, until a certain threshold value (7.5%), the increase of PVP percentage reduced the dope thermodynamic stability, induced faster liquid/liquid demixing and promoted the formation of macrovoids. Further increase of PVP concentration produced a rheological hindrance of the demixing, due to high solution viscosity, which delayed the overall diffusion between components. Simone *et al.* [25] reported similar observations in the production of PVDF hollow fibers. At low concentration (from 6 to 9 wt.%), PVP enhanced the dope instability, and, hence, promoted faster demixing and macrovoids formation (thermodynamic effect). On the contrary, at high concentration (11–15 wt.%), PVP increased the dope viscosity, delayed L/L demixing and reduced macrovoids development (kinetic effect).

The morphology of the PVDF fibers produced in this work suggests a clear predominance of thermodynamic over kinetic effect and can be explained as follows. PVP is known to affect the thermodynamic of the phase inversion process due to its hydrophilicity. The PVP percentage in the dope was very high (35 wt.%); it was out of the range investigated by Simone *et al.* [24] (0–15 wt.%). Such a high additive percentage in the dope strongly increased its instability, thus promoting faster coagulation and enhancing macrovoids growth (thermodynamic effect). It is worth mentioning that the high polymer and PVP percentages in the dope solution (25 and 35 wt.%, respectively) led to very high viscosity at room temperature. According to what observed in literature, this should delay demixing due to kinetic hindrance. However, fibers spinning was carried out at 115 °C, and the dope was visually fluid at the working temperature. Therefore, kinetic hindrance of macrovoids development was not observed.

The inner coagulant is widely recognized as one of the main factors affecting the solvent/non-solvent exchange rate and, thus, hollow fibers morphology. For instance, Sukitpaneenit and Chung [34] reported that, depending on the non-solvent, liquid/liquid and solid/liquid demixing can both take place during PVDF membrane coagulation, due to its semi-crystalline nature. In particular, they observed spherical globules of semi-crystalline PVDF when using “softer” coagulants, as methanol, ethanol or isopropanol. The globular structure, resulting from solid/liquid demixing, could grow only thank to delayed coagulation, which left enough time to induce crystallization. On the other hand, Simone *et al.* [25] reported that, even if slower demixing can be achieved by using aqueous mixtures of solvents as coagulants, the bore fluid composition affected mainly macrovoids formation at the inner layer of PVDF fibers. Furthermore, at high PVP concentration, this effect was less evident.

As reported in Table 1, PVDF fibers were spun using a particularly high PVP percentage in dope solution (35 wt.%). Therefore, the effect of dope composition overcame that of bore fluid on hollow fibers morphology.

Temperature was another factor that surely affected the produced hollow fibers morphology. Indeed, temperature is a key parameter both referring to the dope and the coagulants [35]. Peng *et al.* [36] reported that, due to reduction of dope viscosity, more macrovoids developed in the cross-sectional morphology of Torlon® polyamideimide fibers, when increasing the spinneret temperature. Temperature affected also the interdiffusion between solvent and non-solvent at the fibers walls, thus enhancing the kinetic of phase inversion. As reported in Table 1, PVDF hollow fibers were spun at 115 °C, while, the bore fluid temperature was kept at 50 °C. Spinning and bore fluid temperature reduced viscosity and promoted solvent/non-solvent exchange, thus enhancing demixing and promoting macrovoid development.

It can be concluded that finger-like morphology developed a consequence of fast L/L demixing due to the particular dope composition and to both dope and bore fluid temperatures. All of these factors led to thermodynamic enhancement of demixing, which was predominant over kinetic hindrance.

### 3.2. Fibers Mechanical Properties, Porosity, Pore Size and Pure Water Permeability

The main properties of the prepared PVDF hollow fibers are resumed in Table 2.

**Table 2:** Main properties of PVDF hollow fibers.

Fiber Type	O.D.	I.D.	Thickness	Emod	εbreak	Porosity	Bubble Point	Pore Diameter	PWP *
	(mm)	(mm)	(mm)	(N/mm²)	(%)	(%)	(bar)	(m)	(L/m^2^·h·bar)
A	2.60 ± 0.02	1.44 ± 0.04	0.58 ± 0.03	39.15 ± 2.94	79.71 ± 5.82	88.32 ± 1.23	1.45	0.31	340
B	2.73 ± 0.03	1.65 ± 0.01	0.54 ± 0.02	28.17 ± 4.50	122.12 ± 7.28	90.64 ± 0.21	0.98	0.14	207
C	2.87 ± 0.03	1.69 ± 0.03	0.59 ± 0.03	20.72 ± 0.50	109.87 ± 6.61	91.36 ± 0.79	0.67	0.17	369
D	2.98 ± 0.02	1.74 ± 0.02	0.62 ± 0.02	19.22 ± 1.45	98.55 ± 2.95	91.54 ± 0.34	0.49	0.22	530
E	3.10 ± 0.02	1.72 ± 0.05	0.69 ± 0.03	15.75 ± 1.44	119.41 ± 4.43	92.10 ± 0.65	0.48	0.12	-
F	2.79 ± 0.05	1.36 ± 0.06	0.71 ± 0.05	17.40 ± 1.32	122.90 ± 5.64	91.25 ± 0.39	0.37	0.14	345

* The standard deviation on PWP measurements is less than 5% in all cases.

Looking at fiber dimension, the fact that inner diameter shows clear dependence on the bore fluid composition and flowrate can be noticed. In particular, the hollow fibers’ I.D. increases, going from fiber type A to type D, with the bore fluid flowrate (and decreases again from fiber type E to type F for the opposite reason, see Table 1). Furthermore, comparing fibers type C and type F, it is evident that, for the same flowrate, the I.D. is larger for fibers spun using water/solvent mixture as bore fluid. These results are in agreement with literature. Tasselli *et al.* [37] observed that solvent percentage in the bore fluid affected the degree of fiber inflation, normally caused by the bore fluid injection. Higher solvent percentage induced a delayed onset of demixing, resulting in a softer skin at the inner surface, easier to inflate. In addition, Simone *et al.* [38] reported that the inner diameter of fibers produced using “softer” inner coagulants enlarged more with the increase of the bore fluid flowrate, due to the different coagulation rate of fibers inner wall.

Fibers were characterized by reduced mechanical strength, reflected by both low Young's modulus and tensile stress at break. At the same time, the produced fibers showed very high porosity, roughly comprised in the range 88%–92%. Both characteristics can be explained taking into account fibers finger-like morphology. Figoli *et al.* [27] reported that the mechanical properties of PVDF hollow fibers were clearly connected to their morphology, while porosity showed typical trade-off with mechanical properties: in general, fibers showing higher porosity showed also reduced Young’s Modulus. Data reported in Table 2 are in agreement with literature and consistent with the morphology described in section 3.1. The big cavities observed in fiber structure represented weak points, while they increased the void fraction very much. Therefore, a typical trade-off between void fraction and mechanical properties was observed.

The bubble point and average pore size of the PVDF hollow fibers are illustrated in Table 2. It can be noticed that, in general, the produced fibers showed an average pore diameter in the range of microfiltration (0.12–0.31 m). The average pore size, in general, is larger for fibers spun using water/solvent mixture as inner coagulant, in agreement to what observed in literature. Simone *et al.* [38] reported that the maximum of the pore size distribution of PVDF hollow fibers slightly shifted towards higher values for fibers spun using “softer” inner coagulants. Similarly, Drioli *et al.* [26] reported that fast solvent/nonsolvent exchange could result in small pores, while slower coagulation promoted the formation of larger pores. In addition, Liu *et al.* [39] observed that faster solvent/nonsolvent exchange resulted in smaller pores, while the initiated nuclei got the chance to grow and develop larger pores before phase separation occurred, if coagulation was delayed.

The water permeability data were in agreement with typical values of microfiltration membranes. Results related to the PWP of hollow fibers type E are not reported due to some defects, which compromised the measurements. Hollow-fibers type E, as can be noticed from the low Young’s modulus value reported in Table 2. Furthermore, with respect to fiber type D, the pore size distribution is less sharp. Among the other fibers, the type D showed the highest PWP value (about 530 L/m^2^·h·bar). Based on the overall characterization results and PWP measurements, hollow fibers type D were selected and used in the orange press liquor processing.

### 3.3. Performance of HF Membranes in the Treatment of Orange Press Liquor

#### 3.3.1. Effect of TMP on Permeate Flux

**Figure 2:**
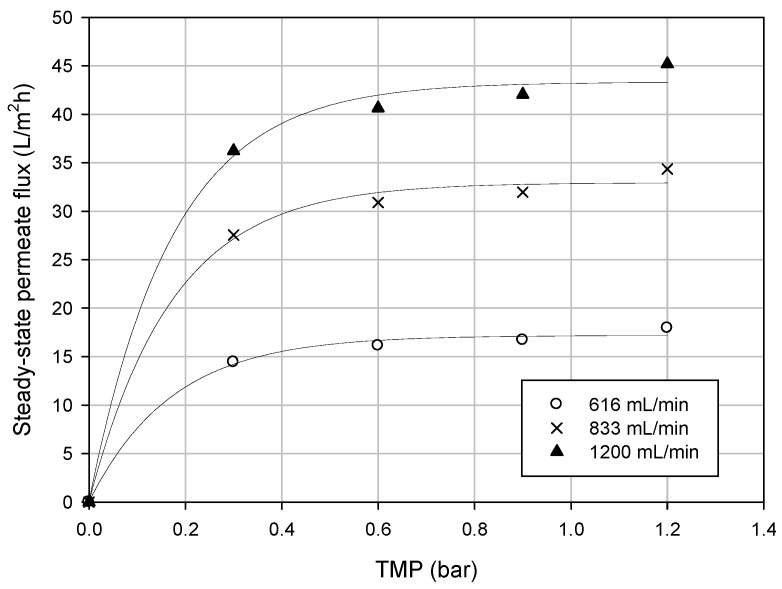
Effect of transmembrane pressure on steady-state permeate flux at different feed flow rate (operating temperature, 25 °C).

The influence of TMP on the steady-state permeate flux was studied at a constant values of temperature (25 °C) and different feed flow rate (616, 833 and 1200 mL/min). As illustrated in Figure 2, a linear relationship was observed between the permeate flux and the applied TMP at low pressure. As the pressure was increased, permeate flux showed a deviation from a linear flux-pressure behavior and it became independent of pressure. In these conditions a limiting flux was reached at a TMP value of about 0.6 bar and any further pressure increase determines no significant increase of the permeate flux.

The existence of a limiting flux can be attributed to the concentration polarization phenomenon that arises as the feed solution is convected towards the membrane where the separation of suspended and soluble solids from bulk solution takes place. According to the gel polarization model, when the rejected particles start to deposit on the membrane surface, the rate of increase in flux decreases. Further increases in pressure determine an increase of the thickness of the particle layer without a corresponding increase in flux. The formation of a viscous and gelatinous-type layer is responsible for an additional resistance to the permeate flux in addition to that of the membrane. The gel-polarized layer is assumed to be dynamic. Higher flow rates tend to remove the deposited material reducing the hydraulic resistance through the membrane, leading to higher mass transport coefficients [40,41]. In particular, at a TMP of 0.6 bar, an increase of the steady-state permeate flux from 11.7 to 29.4 L/m^2^·h was observed when the feed flow rate was increased from 616 to 1200 mL/min. A similar behavior was also observed in the clarification of kiwi fruit juice by using poly(ether ether ketone) HF membranes [21].

#### 3.3.2. Experimental Runs According to the Batch Concentration Mode

**Figure 3:**
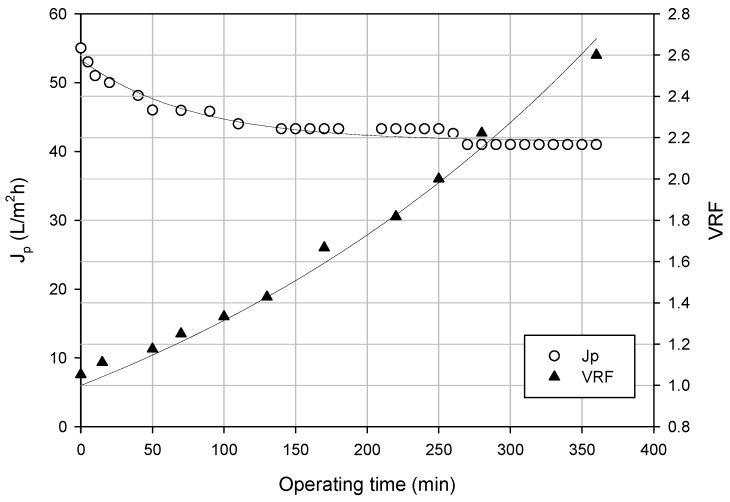
Clarification of orange press liquor by PVDF HF membranes according to the batch concentration mode. Time course of permeate fluxes and VRF (operating conditions: TMP, 0.6 bar; feed flow rate, 1200 mL/min; T, 25 °C).

Figure 3 shows the productivity of the HF membranes when the press liquor is processed at a TMP of 0.6 bar, a feed flow rate of 1200 mL/min and a temperature of 25 °C according to a batch concentration configuration. The initial permeate flux of about 55 L/m^2^·h decreased gradually with the operating times by increasing the volume reduction factor (VRF, defined as the ratio between the initial feed volume and the volume of the resulting retentate) due to concentration polarization and fouling phenomena up to reach a steady-state value of 41 L/m^2^·h.

The productivity of the selected membranes resulted higher than those reported in literature in the clarification of different fruit juices. In particular, Carvalho *et al.* [42] evaluated the performance of different polymeric ultrafiltration (UF) and MF membranes in the clarification of pineapple juice; for PVDF tubular membranes, permeate fluxes measured at an operating pressure of 1.5 bar (higher than that used in this work) were of about 36 L/m^2^·h. Mirsaeedghazi *et al.* [43] obtained permeate fluxes at steady state conditions varying from 3 to 6 L/m^2^·h in the clarification of pomegranate juice with PVDF membranes having pore size of 0.22 and 0.45 m, respectively.

**Figure 4:**
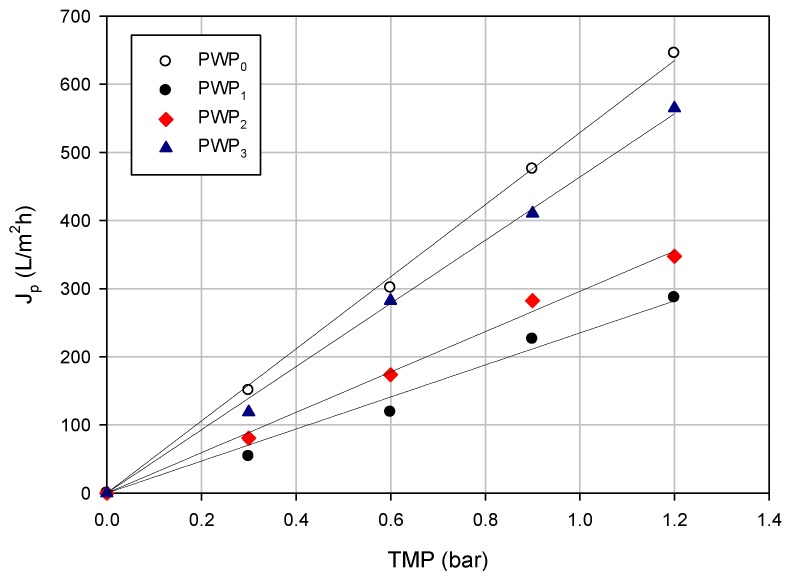
Pure water permeability of PVDF FH membranes before and after washing cycles (PWP_0_, initial pure water permeability; PWP_1_, pure water permeability after press liquor filtration; PWP_2_, pure water permeability after cleaning with water; PWP_3_, pure water permeability after chemical cleaning).

Figure 4 shows the water permeability of the selected membranes after each cleaning cycle. The initial water permeability (PWP_0_), of about 530 L/m^2^·h·bar), was reduced to 235 L/m^2^·h bar after the treatment of the orange press liquor. The fouling index calculated according to Eq. 3 was of 55.6%. A first cleaning with distilled water permitted to recover about 56% of the initial water permeability (296 L/m^2^·h·bar), due to the removal of the reversible polarized layer; a chemical cleaning with a 50 ppm sodium hypochlorite solution (at 40 °C for 60 min) permitted to recover about 87% of the water permeability. Therefore, the incomplete recovery of water permeability can be attributed to an irreversible component of fouling.

#### 3.3.3. Analytical Results

As reported in literature, peels of fruits are major sources of natural antioxidants [6]. In particular, by referring to citrus fruits, previous studies have shown the presence of higher contents of phenolic compounds in the peels in comparison to the juice [44]. The recovery of these compounds and their reuse as natural antioxidants is of great interest to valorize these by-products and offer new opportunities for the formulation of products of interest in food (dietary supplements and functional foods production), pharmaceutical (products with antibacterial, antiviral, anti-inflammatory, antiallergic and vasodilatory action) and cosmetic industry.

Table 3 shows the influence of the clarification treatment with the PVDF hollow fibers on orange press liquor composition. Experimental results showed that suspended solids were completely removed by the HF membranes, while total soluble solids and pH remained quite unchanged in comparison to the unclarified liquor.

**Table 3:** Physico-chemical properties of orange press liquor treated by PVDF hollow fibers.

Parameters	Feed	Permeate	Retentate
Suspended solids (%)	8.3 ± 0.2 ^a^	-	9.0 ± 0.5 ^a^
TSS (°Brix)	18.0 ± 0.1 ^a^	17.8 ± 0.2 ^a^	18.2 ± 0.4 ^a^
pH	3.5 ± 0.2 ^a^	3.5 ± 0.1 ^a^	3.4 ± 0.4 ^a^
TAA (mM Trolox)	21.4 ± 3.5 ^a^	21.1 ± 3.7 ^a^	18.4 ± 2.2 ^a^
Total polyphenols (as GAE) (ppm)	1217.3 ± 57.0 ^a^	1167.0 ± 30.6 ^a^	1346.6 ± 13.8 ^b^
Neohesperidin (ppm)	20.70 ± 0.41 ^a^	19.50 ± 0.39 ^a^	17.00 ± 0.34 ^b^
Hesperidin (ppm)	18.40 ± 0.36 ^a^	17.40 ± 0.34 ^b^	19.20 ± 0.38 ^a^
Naringin (ppm)	5.54 ± 0.11 ^a^	5.51 ± 0.11 ^a^	5.82 ± 0.11 ^b^

For each parameter, mean values within a row with different letters are significantly different at *p* < 0.05.

**Figure 5:**
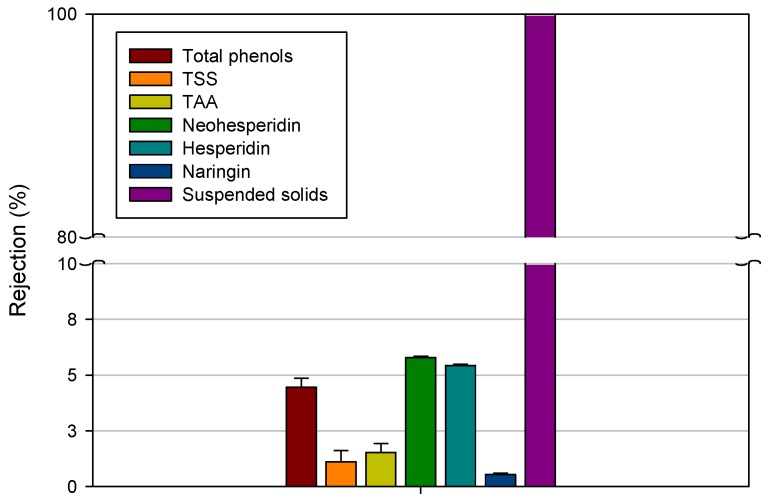
Rejection of PVDF HF membranes towards investigated compounds of orange press liquor.

Analyses of phenolic compounds according to the Folin–Ciocalteu method revealed that these compounds were very well preserved in the clarified fraction. The prepared membranes exhibited a rejection of 4.46% towards these compounds (Figure 5). Ultrafiltration membranes with molecular weight cut-off of 25 and 100 kDa were found to exhibit higher rejections towards phenolic compounds (40 and 13%, respectively) of olive mill wastewaters according to their lower pore size [45].

Neohesperidin was the major flavonoid detected in the feed solution, with concentration of 20.7 ppm, followed by hesperidin (18.4 ppm) and naringin (5.54 ppm). All of these compounds were also were preserved in the permeate streams. Indeed, rejections of the HF membranes towards these compounds were in the range 0.6–5.8%. A low retention of PVDF membranes towards phenolic compounds was also observed by Galanakis *et al.* [46] in the ultrafiltration of winery sludge in comparison with PSU membranes. In particular, the PVDF membrane presented a lower retention towards flavonoids and anthocyanins despite its lower molecular weight cut-off (1 kDa) in comparison to PSU membranes (100 and 20 kDa). This behavior can be explained assuming the different chemical nature of both polymers. Indeed, PSU contain oxygen and sulfur dioxide subunits which provide it with a hydrophilic character. PVDF, on the contrary, is made of alternating units of CH_2_ and CF_2_ conferring a hydrophobic nature to the material. Therefore, the hydrophilic subunits of PSU polymer could be more prone to create hydrogen bonds and Van-der-Waals interactions with the hydroxyl groups of flavonoids with consequent adsorption of these components at membrane surface and fouling phenomena. On the other hand, PVDF HF membranes are less susceptible to hydrogen bonds and van der Waals’ interactions and, consequently, more resistant to fouling and highly permeable to flavonoids.

The total antioxidant activity is a well established biomarker, which indicates beneficial biological effects of plant derived substances. In this work, the antioxidant capacity was evaluated with the ABTS *in vitro* assay. The high value of the TAA (21.4 ± 3.5 mM of Trolox) of orange press liquor is mainly attributed to the presence of different bioactive compounds and particularly of flavonoids that present similar radical scavenging activity with Trolox [47]. In addition, for the TAA, the produced hollow fibers showed a low rejection (1.4%) in agreement with the recovery of flavonoids in the clarified liquor.

## 4. Conclusions

PVDF hollow fibers have been prepared by the dry/wet spinning technique. A high amount of PVP was added to the polymeric dope, ensuring high dope viscosity and, hence, good mechanical resistance of the produced fibers. Moreover, the treatment with sodium hypochlorite allowed removal of the residual PVP, ensuring high fibers permeability.

All fibers have been characterized in terms of morphology, mechanical properties, pore size and pure water permeability. On the basis of characterization results fibers exhibiting pore size of about 0.2 m, porosity of about 90% and highest values of pure water permeability (530 L/m^2^·h·bar) have been selected and used to clarify orange press liquor.

At steady conditions, permeate fluxes of about 40 L/m^2^·h were measured when the liquor was processed in optimized operating conditions according to a batch concentration configuration. The clarified liquor is characterized by high antioxidant activity due to the low rejection of the membranes towards phenolic compounds. In particular, a 0.6–5.8% rejection towards flavanones, highly recognized for their pharmacological properties, has been detected.

These results confirm that the prepared membranes represent a valid approach for producing intermediate products from orange press liquor, which can be reused to develop innovative formulations for functional foods and pharmaceutical products.
